# Technology and cardiovascular diseases in the era of COVID‐19

**DOI:** 10.1111/jocs.15096

**Published:** 2020-10-10

**Authors:** Amer Harky, Ahmed Adan, Malak Mohamed, Asha Elmi, Thomas Theologou

**Affiliations:** ^1^ Department of Cardiothoracic Surgery Liverpool Heart and Chest Hospital Liverpool UK; ^2^ Department of Integrative Biology, Faculty of Health and Life Sciences University of Liverpool Liverpool UK; ^3^ Liverpool Centre for Cardiovascular Science, Liverpool Heart and Chest Hospital University of Liverpool Liverpool UK; ^4^ Department of Cardiac Surgery Alder Hey Children Hospital Liverpool UK; ^5^ Faculty of Health and Life Sciences, School of Medicine University of Liverpool Liverpool UK; ^6^ Faculty of Medicine and Health, School of Medicine University of Leeds Leeds UK

**Keywords:** coronavirus, heart, information technology, vessels

## 1 INTRODUCTION

The global coronavirus disease 2019 (COVID‐19) pandemic has had unprecedented effects on healthcare systems worldwide. Hospitals have been under intense strain as a result of the surge in COVID‐19 cases, and there has been a stark reduction in the inpatient provision of cardiovascular care.^1^ An alternative to inpatient service provision is increased use of digital healthcare tools.^2^ The rapid advancements in technology and the rise in popularity of mobile devices in recent years meant that telemedicine is an increasingly viable option.^3^ The Guidance from the National Health Service in the United Kingdom supports the use of digital consultations where possible to reduce the number of in‐person appointments in an attempt to reduce the risk of COVID‐19 spread and care provision for those who need to be under regular follow‐up.^4^

The utilization of digital technology and telemedicine in healthcare can come in different forms including but not limited to telephone and video consultations.^3^ These tools can be used not only to follow‐up cases but also to diagnose new conditions in patients through direct patient interaction over video consultation and assessment. While primary care has incorporated telemedicine into clinical practice in recent times with regular telephone consultations and home monitoring of patients, this may be slightly more difficult when used for patients with cardiovascular disease due to increased complexity.^5^ However, due to the current COVID‐19 pandemic and the resultant strain on healthcare systems worldwide, innovative solutions are needed in all aspects of patient care including in secondary and tertiary care.

Patients with cardiovascular disease can benefit from the application of telemedicine in the form of remote consultations, monitoring of clinical parameters, and remote diagnostic investigations. A randomized controlled trial evaluating the effectiveness of telephone consultations for patients with heart failure found that there were no significant differences in the rate of hospitalizations, readmission rate, or mortality between patients managed using the telephone approach or usual face‐to‐face care.^6^

Similarly, the home or hospital in heart failure study, a multinational randomized controlled trial assessing the feasibility of home telemonitoring (HT) of clinical parameters in decreasing cardiac events, found that HT is feasible and has high compliance among patients, and can potentially reduce clinical instability.^7^ Another potential use of telemedicine in cardiovascular care is the remote provision of cardiac rehabilitation, which includes patient education and supervised exercise delivered by telephone or video conferencing, and this approach can be highly effective.^8^ Telemedicine can also be utilized in the “forward triage” of patients, using smartphone devices or computers to screen patients before they arrive at the emergency department. This is of particular importance given the current COVID‐19 pandemic, as patients with high‐risk features for the virus can be isolated quicker upon arrival, therefore minimizing exposure to other patients and staff.^9^

## 2 THE ROLE OF DIGITAL HEALTHCARE IN PERIOPERATIVE PERIOD

As patients with cardiovascular diseases that are requiring surgery are at a higher risk of contracting the COVID‐19 infection, with higher morbidity and mortality than the general population,^10^ telemedicine and digital healthcare can play a valuable role in their preoperative and postoperative care; in addition to long‐term follow‐up and possibly reducing the time needed to be spent at hospital amidst the pandemic. Preoperatively, it is possible to triage patients and conduct preoperative assessments and investigations remotely.^11^ Echocardiograms can be analyzed remotely during real‐time consultations to triage cardiac surgery patients,^12^ and arrhythmias can be detected by utilizing a smartwatch or remote devices.^13^ In addition, teleconsultations of angiograms have proven successful in diagnosing and referring patients for procedures, including coronary artery bypass graft surgery.^12^

A limitation of this type of remote technology during the COVID‐19 pandemic is that its use depends on imaging equipment and trained technicians performing the scans before a remote specialist can interpret them, necessitating contact between patients and healthcare professionals.^11^ Tele‐electrocardiogram (ECG) home‐monitoring, on the other hand, is more appropriate in the context of the current pandemic, using patient‐friendly mobile phone applications to allow the transmission of ECG traces of surgical candidates directly to specialists for analysis.^11^ A system of wearable sensors has also been developed, which utilizes Bluetooth to transmit information to mobile phones through a web interface, allowing physicians to monitor heart rate, blood pressure, and temperature remotely in real‐time.^14^

Postoperatively, the use of smartphones, web applications, video conferencing, and image transmission has facilitated the identification of surgical complications, including surgical site infections, allowing patients to receive appropriate and timely care.^11^ While McElroy et al^15^ showed no significant differences in readmission rates following cardiac surgery for patients who received digital health kits compared to those who did not, they demonstrated that such tools can be used to manage cardiac surgical patients postoperatively; this may be particularly helpful in reducing the need for hospital‐based outpatient follow‐up amidst the current pandemic. Moreover, video consultations in addition to remote telemonitoring have proven effective in ambulatory follow‐up after implantation of a cardiac device,^16^ and telemonitoring of physiological data from cardiac resynchronization defibrillators allows the identification of cardiac complications, including decompensations.^11^

Longer term telemedicine also provides an alternative to in‐hospital cardiac rehabilitation programs.^11^ At‐home exercise equipment and tele‐ECG monitoring, paired with regular telephone and videoconferencing with healthcare professionals, allow patients to be monitored for complications and to complete their physiotherapy regimens, while reducing hospital contact.^17^, ^18^ The Fit@Home study demonstrated similar short‐term improvements in exercise capacity and quality of life with telemonitoring‐guided home‐based rehabilitation programs compared to center‐based approaches.^19^ However, the success of these interventions may not apply to high‐risk or unstable patients, who still require in‐hospital facilities, and patients without support at home.^11^ For patients who can be followed up remotely, however, telemedicine has shown similar clinical effectiveness and patient satisfaction to face‐to‐face consultations.^11^ It also has a role in health promotion and prevention of cardiac events, via weight loss, smoking cessation, and glycaemic control programs, all of which can be achieved using phone calls, text or web‐based messaging, video consultations, and monitoring devices.^20^, ^21^

## 3 ADVANTAGES AND DISADVANTAGES OF DIGITAL CARDIOVASCULAR CARE

The role of digital healthcare has become increasingly prevalent amid the current climate in all aspects of medicine, particularly in patients with cardiovascular disease.^22^ The advantages and disadvantages of telehealth have been explored and cited in various literature including in the treatment, diagnosis, and management of various cardiovascular conditions. The effectiveness and reduction in hospital cases of coronavirus by incorporating telehealth has been noted to be a favorable factor for management of cardiovascular conditions. A study developed a triaging system which involved following up patients with uncomplicated diabetic foot ulcers by telemedicine after outpatient evaluation found an adequate management of diabetic foot ulcers which led to no cases of hospital virus exposure for the patients and did not compromise patient care.^23^

In addition, telehealth can be used with high accuracy to detect cardiovascular conditions. A study which assessed the accuracy of smartphone technology in detecting atrial fibrillation (AF) with physician interpretation, using a kardia mobile cardiac monitor, found a 96.6% sensitivity and 94.1% specificity for AF detection compared with physician interpreted ECGs.^24^ Due to the nature of AF meaning that it can be subclinical, and, therefore, undetected in ECGs conducted in a healthcare setting, this digital healthcare tool can be used to help reduce the number of patients who do not get diagnosed, and, therefore, improve patient outcomes.

Telemedicine has an increasing importance due to the current climate in also helping improve long‐term health outcomes, increase attrition rate, and remove the barriers of cardiac rehabilitation programs for those in need, which has been done with success. A prospective study conducted during the COVID‐19 pandemic found that the participation rate increased in heart failure patients who were in the remote cardiac rehabilitation program, and the emergency readmission rate within 30 days was lower in the remote (*n* = 30) compared to the nonremote cardiac rehabilitation group (*n* = 137).^25^ This helps us to improve the lives of those with chronic health conditions who may not be able to undergo traditional cardiac rehabilitation due to COVID‐19.

However, there are drawbacks to digital healthcare which have been highlighted by the literature. First, there is the need for understanding from both physician and patient of the digital tools in order for telemedicine to work. A prospective survey which explored patient and doctor experience with the use of virtual visits in the care of patients with arrhythmia found although both patient and physician experience was largely positive, 9% of participants were unable to take part in the study due to technical difficulties,^26^ highlighting the subsequent negligence which may arise if digital health was the only option for patients, such as the elderly, or those with learning difficulties who may find telehealth complex. Another disadvantage of digital cardiovascular care is its inability to be an adequate replacement for physician analysis, as the General Medical Council guidance states that telemedicine should only be considered in situations where there is a prior knowledge of the patient beforehand and the condition is not complex.^27^ Therefore, this would not be useful in patients who have more challenging needs which would require a face‐to‐face consultation to give a good level of care. Although the increasing digital cardiovascular care given to patients during this pandemic has been largely positive in improving patient care and treatment, the decision of its use has to be tailored to each individual patient in order for it to be beneficial.

## 4 POTENTIAL USES OF DIGITAL OR ROBOTIC SYSTEMS IN CARDIOVASCULAR CARE

The potential uses of digital and robotic systems in cardiovascular care have been explored by the scientific community on both a theoretical basis and with practical clinical studies. The Xiao et al.^28^ study propose a wearable heart rate monitor for sports, which transmits real‐time data into personal computer or mobile phone, allowing long‐term ECG and pulse waves signals to be documented.^28^ This could be used for monitoring conditions such as long‐standing arrhythmias, to analyze effectiveness of treatment and severity of the condition. Areas of innovation noted in a journal article on management of aortic stenosis have suggested increased use of wearable and remote devices to assess patient performance and vital signs and devices for facile cardiac assessment during the COVID‐19 era.^29^

Similarly, cardiovascular surgery and the potential use of robotic‐assisted technology has been reviewed to improve accuracy and precision and reduce harm to the interventionist. A case report of a 73‐year‐old male with severe carotid artery stenosis where a robotic‐assisted balloon angioplasty and stent placement using the CorPath GRX Robotic system was used in a high‐risk surgical patient found no complications after the procedure and the patient was discharged on aspirin, clopidogrel, and rivaroxaban. At 3‐month follow‐up, the patient was asymptomatic and with no longer term complications. The potential use of robotic systems on a large scale is beneficial as it can help to reduce exposure of fluoroscopic radiation, as well decreasing complications associated with surgery and anesthesia.^30^

## 5 PERSISTENCY OF DIGITAL HEALTH SERVICE BEYOND COVID‐19

As put by novelist scholar Arundhati Roy, “the pandemic is a portal,”^31^ resulting in a proliferation of digital health technology, facilitating continued access to essential care. With digital health tools being increasingly used to deliver healthcare services while maintaining social distancing, governments and technology actors continue to discuss the permanent integration of such digital tools into postpandemic life.^32^ To achieve this however, concerns regarding social inequalities and barriers to digital health access and literacy require addressing, including social, cultural, and economic factors affecting access to technology, which may put some patients, particularly those from underresourced areas, at a disadvantage.^33^

Information governance is also a concern, with the potential for patient confidentiality and data security to be compromised; digital platforms used for telehealth should therefore, be secure, reliable, and compliant with healthcare regulations.^34^ In particular, many primary care services have switched to virtual delivery, although there is insufficient evidence on the impact of such services on clinical outcomes and quality of healthcare.^35^ To promote the expansion of telehealth in a post‐COVID era, collaborative research into the effectiveness of digital healthcare delivery is necessary.^36^, ^37^, ^38^, ^39^, ^40^ Professional and patient input, paired with government‐supported health technology expansions, is needed^34^ to ensure that such developments do not exacerbate social inequalities and data security issues, but rather, that they improve digital literacy and remove barriers to healthcare.

## CONFLICT OF INTERESTS

The authors declare that there are no conflict of interests.
